# Early Gut Microbiome Alterations in Mild Cognitive Impairment Reflect Changes in Alzheimer Disease

**DOI:** 10.1097/WAD.0000000000000732

**Published:** 2026-06-03

**Authors:** Emilia Brandt, Anne Koivisto, Pedro Pereira, Ella Mustanoja, Petri Auvinen, Toni Saari, Minna Rusanen, Ville Leinonen, Filip Scheperjans, Virve Kärkkäinen

**Affiliations:** * Institute of Clinical Medicine, School of Medicine, University of Eastern Finland, Kuopio, Finland; † NeuroCenter, Kuopio University Hospital, Kuopio; ‡ Department of Neurosciences, Clinicum, Faculty of Medicine; ¶ Institute of Biotechnology; # Institute for Molecular Medicine Finland (FIMM), Helsinki Institute of Life Sciences; ** Clinicum, University of Helsinki; Departments of § Geriatrics; ‖ Neurology, Helsinki University Hospital, Helsinki; †† Geriatric Center, Wellbeing Services County of North Karelia, Joensuu, Finland

**Keywords:** Alzheimer disease (AD), dementia, biomarker, gut microbiome (GM), mild cognitive impairment (MCI)

## Abstract

**Introduction:**

Alterations in the gut-brain axis have been increasingly linked to neurodegenerative diseases, including Alzheimer disease (AD). It remains unclear whether these microbiome changes are already present during early cognitive decline. We examined whether gut microbiome alterations characteristic of AD are detectable in mild cognitive impairment (MCI) and whether these changes follow a similar pattern across the cognitive continuum.

**Methods:**

This case-control study included 78 participants: 37 cognitively healthy controls, 20 individuals with MCI, and 21 individuals with prodromal or mild AD. Cognitive performance was assessed using the CERAD neuropsychological battery, and disease severity was assessed using the Clinical Dementia Rating. Dietary data were collected, and fecal samples were analyzed using 16S rRNA gene amplicon sequencing.

**Results:**

We identified 16 bacterial genera associated with cognitive status. Genera such as *Lacticaseibacillus*, *Raoultella*, and *Buttiauxella* were reduced in AD, with similar decreases already evident in MCI. In contrast, *Anaerovorax* and an unclassified *Comamonadaceae* genus were increased in AD. Several alterations showed a consistent trend from normal cognition through MCI to AD.

**Discussion:**

Gut microbiome alterations characteristic of AD appear already present in early cognitive decline and follow a similar pattern in MCI. These findings support the potential of microbiome profiles as early, noninvasive biomarkers of AD.

Alzheimer disease (AD) is a progressive neurodegenerative disorder in which neuronal loss ultimately leads to severe dementia.^1^ Its pathologic hallmarks include Tau neurofibrillary tangles and beta-amyloid plaques, detectable in blood, cerebrospinal fluid, and through PET imaging.^2^ Beta-amyloid accumulation may progress for up to 2 decades before clinical symptoms emerge.^3^ Early symptoms typically involve mild cognitive deficits, especially episodic memory impairment, followed by a gradual decline in daily functioning as the disease advances from mild to moderate and severe dementia.^1^ Early diagnosis is essential, as emerging therapies and nonpharmacological interventions target the initial stages of the disease to delay progression and support quality of life.^4^

Mild cognitive impairment (MCI) describes a heterogeneous group with minor memory deficits. MCI may be reversible, stable, or progressive, and some individuals develop dementia. The earliest AD stage preceding mild dementia is referred to as AD-MCI, although MCI can also precede other neurodegenerative diseases, such as Parkinson disease.^5^

There is an urgent need for biomarkers that identify the earliest stages of AD. One promising candidate is the gut microbiome (GM), which shows alterations in neurological conditions such as Parkinson disease (PD) and idiopathic normal pressure hydrocephalus (iNPH).^6,7^ The gut-brain axis enables bidirectional communication through pathways including the vagus nerve, neurotransmitters, and other molecular mechanisms.^8^ In PD, specific GM alterations have already been identified.^6^

Although fewer studies exist for AD, evidence suggests that GM composition changes during preclinical stages.^9^ GM alterations may also provide insight into AD pathology. Certain bacteria can affect epithelial tight junctions, enabling bacterial components or neurotoxins to enter systemic circulation,^8^ and some findings indicate that gut bacteria may influence undefined role in beta-amyloid accumulation in the brain.^10^

Our aim was to determine whether individuals with early cognitive impairment (MCI group) exhibit GM changes compared with healthy controls (CO). We also compared the MCI and AD groups and assessed whether specific alterations show a gradual pattern across cognitive decline.

## METHODS

### Study Design, Participants, and Protocol

This case-control study compared gut microbiome composition among individuals with Alzheimer disease (AD), mild cognitive impairment (MCI), and cognitively healthy controls (CO). A total of 78 participants were recruited by the Brain Research Unit at the University of Eastern Finland between 2018 and 2019.

Participants were assigned to 3 groups based on neuropsychological assessment and clinical diagnosis. The CO group included 37 individuals (mean MMSE 28.4±1.5). The MCI group comprised 20 individuals with mild cognitive impairment without dementia (mean MMSE 27.0±1.5). The AD group included 21 individuals diagnosed with prodromal or mild AD before enrollment (mean MMSE 23.9±3.0). Exclusion criteria included diabetes, depression, other neurological disorders such as PD, moderate or severe AD (CDR 2 to 3), and non-AD dementias.

Each participant attended 2 study visits. The protocol included a demographic interview, clinical and neurological examination, and ApoE-ε4 genotyping from venous blood. Cognitive performance was assessed using the Consortium to Establish a Registry for Alzheimer disease neuropsychological battery (CERAD-NB), and disease severity with the Clinical Dementia Rating (CDR) global score.^11,12^

Controls were required to show normal cognitive performance and no functional impairment based on CDR and clinical evaluation. Individuals in the MCI group had preserved daily functioning and were classified according to CERAD-NB performance in the absence of biomarker data.

AD diagnoses were established before enrollment by a neurologist or geriatrician according to the National Institute on Aging-Alzheimer’s Association (NIA-AA) criteria.^13^ AD stage was determined using the CDR global score (0.5 to 1.0). Participants in the AD group underwent neuroimaging (MRI or CT) and laboratory testing for differential diagnosis, and standard clinical treatment was initiated. Biomarker data were not available at the time of recruitment.

### CERAD-NB

Cognitive performance was evaluated using the Finnish version of the CERAD neuropsychological battery (CERAD-NB), which includes the same subtests as the original English version.^12^ The battery consists of the Mini-Mental State Examination (MMSE; range: 0 to 30), the 15-item Boston Naming Test (range: 0 to 15), category fluency for animals, word list learning (range: 0 to 30), word list recall (range: 0 to 10), word list recognition (range: 0 to 20), and constructional praxis (range: 0 to 11). The Finnish version additionally includes delayed recall of constructional praxis (range: 0 to 11) and the clock-drawing test (range: 0 to 6). A CERAD-NB Global Memory Score was calculated by summing word list learning, delayed recall, and recognition (maximum 50 points).

### ApoE Genotyping

ApoE-ε4 is a well-established genetic risk factor for sporadic AD.^14^ Genomic DNA was extracted from venous blood using the QIAamp DNA Blood Mini Kit (QIAGEN). ApoE genotype was determined with TaqMan genotyping assays (Applied Biosystems, Foster City, CA) targeting 2 single-nucleotide polymorphisms (rs429358 and rs7412). Allelic discrimination was performed on the ABI 7000 platform following standard procedures.^15^

### Fecal Sample Collection and DNA Analysis

Participants reported their dietary habits for the week preceding the study visit, including consumption of meat, legumes, vegetables, grains, dairy products, coffee, beer, and alcohol. Information on comorbidities and medications, particularly cardiovascular and metabolic conditions, was also collected.

Fecal samples (FS) were collected at home at least 3 days before the first study visit using containers prefilled with DNA stabilizer (PSP Spin Stool DNA Plus Kit; STRATEC Molecular). Samples were stored in home freezers, transported to the Brain Research Unit on ice, and subsequently stored at –80 °C. Sample collection was recommended to occur as close as possible to the study visit. For transport to Helsinki for sequencing, samples were kept on dry ice.

DNA from fecal samples was extracted using the PSP Spin Stool DNA Plus Kit (STRATEC Molecular). Detailed extraction and sequencing procedures have been described previously.^7^ In brief, the 16S rRNA V3-V4 regions were amplified by PCR and sequenced on the Illumina MiSeq platform (v3 600-cycle kit), producing 326-bp forward and 278-bp reverse reads. Barcodes were selected using BARCOSEL.^16^ Primer trimming was performed with Cutadapt v3.7,^17^ and quality filtering, ASV inference, and taxonomic classification were conducted using DADA2 v1.18.0 in R v4.0.5.^18,19^

### Statistical Analyses

IBM SPSS Statistics was used to analyze CERAD-NB subtests and demographic variables. Group differences in categorical variables, such as ApoE-ε4 carrier status and gender, were assessed with χ^2^ tests. For continuous variables, including age, education, and CERAD-NB scores, one-way ANOVA with Bonferroni-corrected post hoc comparisons was applied.

Alpha diversity was assessed at the ASV level using Wilcoxon rank-sum tests with a significance threshold of *P*≤0.05. Beta diversity was analyzed in R using distance-based multivariate methods (PERMANOVA, vegan package) to evaluate the effects of clinical and technical variables on sequencing data.^7,19,20^ On the basis of PERMANOVA results, differential abundance models were adjusted for arrhythmia and statin use. Differential abundance analyses were performed in R using the DESeq. 2 package.^21^

## RESULTS

### Demographics, Bowel-Related Factors, and Dietary Habits

In the AD group, 78.9% of participants were APOE-ε4 carriers, compared with 37.8% in the CO group and 35% in the MCI group. The difference was significant between CO and AD (*P*≤0.05). No significant group differences were observed in comorbidities, bowel-related factors, or dietary habits (Table 1).

**TABLE 1:** Demographic Data and the Bowel-Related Factors of the Study Participants

	CO	MCI	AD			
	37	20	21	*P*_CO-MCI_	*P*_CO-A_	*P*_MCI-AD_
Age (y)	71.0 (5.1)	72 (6.4)	71.1 (6.9)	1.00	1.00	1.00
Women (%)	54.1 (20)	45.0 (9)	61.9 (13)	1.00	1.00	0.86
Education (y)	12.6 (4.3)	11 (3.7)	12.5 (4.2)	0.39	1.00	0.57
ApoE-ε4 carrier	37.8 (14)	35 (7)	78.9 (15)	1.00	**0.04**	0.055
BMI (kg/m^2^)	25.4 (2.85)	25.2 (1.50)	24.9 (3.97)	1.00	1.00	1.00
Bowel-related factors						
Lactose intolerance	27.0 (10)	5.0 (1)	9.5 (2)	0.10	0.25	1.00
Celiac disease	8.1 (3)	5.0 (1)	0	1.00	0.56	1.00
Inflammatory bowel diseases	2.7 (1)	0	4.8 (1)	1.00	1.00	1.00
Irritary bowel syndrome	5.4 (2)	10.0 (2)	4.8 (1)	1.00	1.00	1.00
Antibiotics (1 mo)	2.7 (1)	10.0 (2)	0	0.53	1.00	0.30
Dietary habits						
Meat	1 (67.6)	1 (55.0)	1 (42.9)	0.19	0.60	1.00
Vegetables	3 (43.2)	2 (50.0)	3 (42.9)	0.07	0.29	1.00
Alcohol	0 (78.4)	0 (70.0)	0 (76.2)	1.00	1.00	1.00

Demographic values are presented as means and SDs, except for gender, ApoE ℇ4 carrier status, and bowel-related factors, which are shown as percentages and the number of participants. Diets are presented as medians on a scale: 0<1 portion per week, 1≥1 portion per week, 2≥1 portion per day, 3=2 to 3 portions per day.

AD indicates Alzheimer disease; CO, control; M, mean; N, number of participants.

Bolded *P*-values represent significant differences between groups (*P*≤0.05).

### CERAD-NB

Three CERAD-NB subtests (verbal fluency, word list learning, and word list recognition) and the global memory score differed significantly between CO and MCI (*P*≤0.02). CO and AD differed significantly in all nine subtests (*P*≤0.02). MCI and AD differed in 5 subtests: MMSE, word list learning, word list savings, constructional praxis, and the global memory score (Table 2).

**TABLE 2:** CERAD-NB Test Results

	CO	MCI	AD	*P*_CO-MCI_	*P*_CO-AD_	*P*_MCI-AD_
CERAD-NB (max score)						
Verbal fluency (animals)	25.1 (7.3)	19.5 (4.6)	15.9 (6.3)	0.010	**<0.001**	0.241
Boston naming test (15)	13.4 (1.7)	12 (3.2)	11.1 (3.3)	0.209	**0.007**	0.806
MMSE (30)	28.4 (1.5)	27 (2.0)	23.9 (3.0)	0.055	**<0.001**	**<0.001**
Word list learning (30)	23.1 (3.1)	17.8 (3.9)	13.3 (2.2)	**<0.001**	**<0.001**	**<0.001**
Word list savings (%)	95.1 (10.6)	75.5 (48.3)	38.6 (29.9)	0.062	**<0.001**	**0.001**
Word list recognition (%)	98.2 (3.6)	85.3 (24.2)	74.8 (11.6)	**0.003**	**<0.001**	0.052
Constructional praxis (11)	10.0 (1.3)	8.3(2.5)	6.0 (3.9)	0.065	**<0.001**	**0.017**
Constructional praxis savings (%)	95.1 (10.1)	85.1 (19.8)	60.8 (39.3)	0.424	**<0.001**	0.005
Clock drawing (6)	5.5 (0.6)	4.8 (1.3)	4.2 (1.6)	0.06	**<0.001**	0.29
Global memory score (30)	27.8 (1.8)	23.1 (2.9)	16.9 (3.3)	**<0.001**	**<0.001**	**<0.001**

AD indicates Alzheimer disease; CERAD-NB, the Consortium to Establish a Registry for Alzheimer disease neuropsychological test battery; CO, control; M, mean; MCI, mild cognitive impairment; MMSE, Mini-Mental State Examination; N, number of participants.

Bolded *P-*values represent significant differences between groups (*P*≤0.05).

### Differences in the Gut Microbiome

**FIGURE 1:**
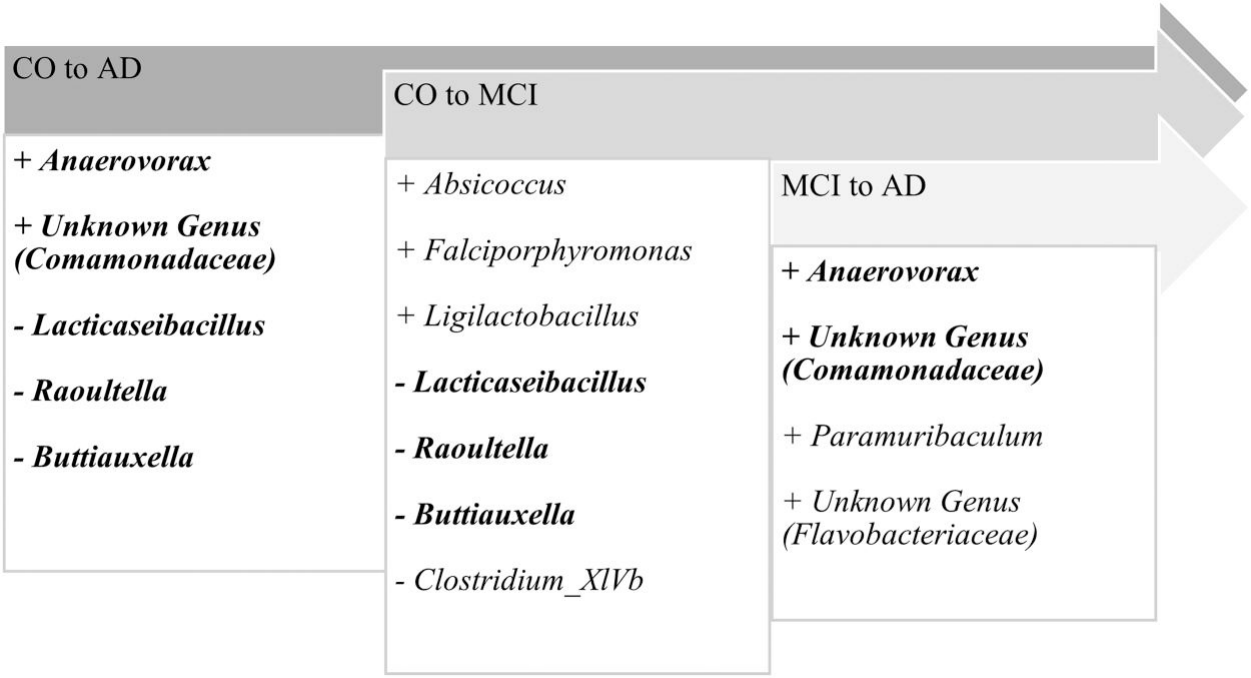
Statistically significant differences in bacterial genera between study groups. Regarding the unknown genera, the closest detectable taxonomical level, the family, is stated in parentheses. Similar changes are shown in bold. “+” indicates increased abundance in the second group relative to the first group; “-”, decreased abundance in the second group relative to the first group; AD, Alzheimer disease; CO, healthy control group; MCI, mild cognitive impairment.

**FIGURE 2:**
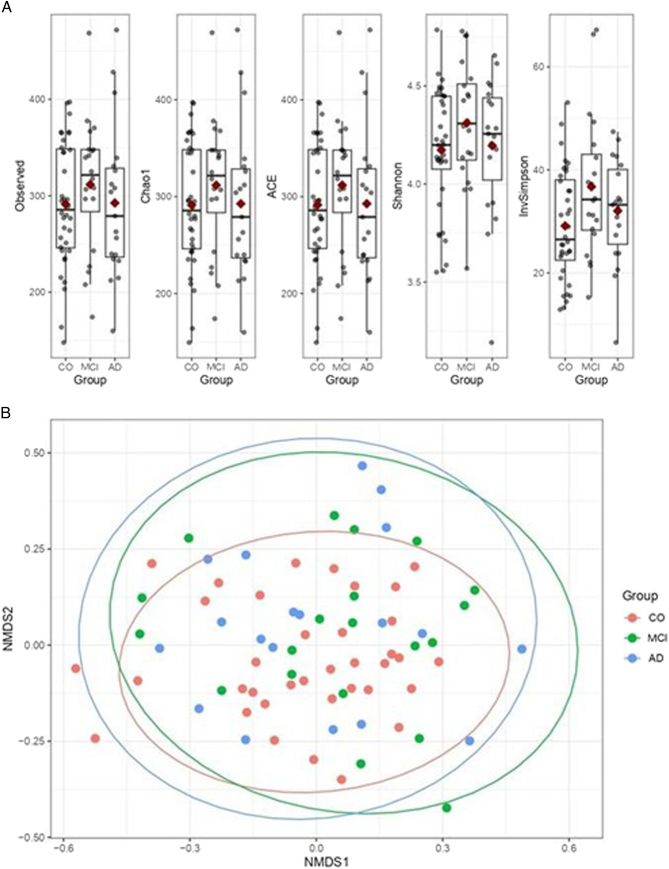
A, Alpha-diversity plot by diversity index and group using ASV level data. Statistically significant differences in the Shannon and Inverse Simpson indices between all groups. Differences in observed, ACE and Chao1 indices between CO and MCI and between MCI and AD, but not between CO and AD. B, Beta-diversity NMDS plot based on Bray-Curtis distance. No significant differences in beta diversity between the groups. ACE indicates an estimated richness index; AD, Alzheimer disease; Chao1, an estimated richness index; CO, healthy control group; InvSimpson, inverse Simpson, richness and evenness index; MCI, mild cognitive impairment; NMDS, nonmetric multidimensional scaling; Observed, observed richness; Shannon, richness and evenness index.

Comparative analyses of gut microbiome genera were conducted across the CO, MCI, and AD groups (Table 3, Fig. 1). We observed group-level differences in alpha diversity across 5 diversity indices, but no significant differences in beta diversity (Fig. 2).

**TABLE 3:** The Comparative Genera Changes Between Groups

Genus	Groups	Log_2_ Fold Change	*P*_adj_
	CO → AD		
*Anaerovorax*	**+**	2.42×10^-7^	0.00101
*Unknown genus, Comamonadaceae family*	**+**	5.61×10^-8^	0.00101
*Enterobacter*	**−**	−2.95×10^-7^	1.66×10^-10^
*Absicoccus*	**−**	−1.91×10^-8^	0.00101
*Buttiauxella*	**−**	−3.78×10^-8^	0.000334
*Raoultella*	**−**	−6.13×10^-8^	5.07×10^-12^
*Lacticaseibacillus*	**−**	−9.32×10^-8^	7.18×10^-10^
	CO → MCI		
*Falciporphyromonas*	**+**	2.70×10^-7^	4.39×10^-9^
*Absicoccus*	**+**	1.06×10^-7^	0.000583
*Ligilactobacillus*	**+**	3.64×10^-9^	9.94×10^-6^
*Clostridium XlVb*	**−**	−6.24×10^-7^	0.00160
*Megasphaera*	**−**	−1.56×10^-7^	0.0109
*Limosilactobacillus*	**−**	−1.44×10^-7^	2.37×10^-5^
*Lacticaseibacillus*	**−**	−1.03×10^-7^	1.39×10^-12^
*Raoultella*	**−**	−7.23×10^-8^	3.51×10^-13^
*Buttiauxella*	**−**	−2.80×10^-8^	3.43×10^-5^
	MCI → AD		
*Clostridium XlVb*	**+**	6.95×10^-7^	0.0237
*Anaerovorax*	**+**	1.90×10^-7^	0.00730
*Paramuribaculum*	**+**	1.70×10^-7^	6.12×10^-8^
*Unknown genus, Flavobacteriaceae family*	**+**	9.23×10^-8^	0.000264
*Unknown genus, Comamonadaceae family*	**+**	5.09×10^-8^	0.00752
*Unknown genus, Sutterellaceae family*	**−**	−1.57×10^-7^	5.36×10^-6^
*Cetobacterium*	**−**	−6.51×10^-8^	0.000264
*Raoultella*	**−**	−3.64×10^-8^	1.93×10^-6^

“+” indicates increased abundance in the second group relative to the first group; “-“, decreased abundance in the second group relative to the first group; log2 Fold change, effect size measured as fold change and expressed in logarithmic scale (base 2); *P*_adj_, adjusted *P*-value (*P*≤0.05).

AD indicates Alzheimer disease; CO, control; MCI, mild cognitive impairment.

When comparing the changing genera between the CO and the AD groups, the amounts of *Anaerovorax* and an unidentified genus of the Comamonadaceae family increased, while *Enterobacter, Absicoccus, Buttiauxella, Raoultella*, and *Lacticaseibacillus* decreased significantly in the AD group. In addition, when comparing the changing genera between the CO and MCI groups, we detected an increase in *Falciporphyromonas, Absicoccus*, and *Ligilactobacillus* in the MCI group. The significantly decreased genera in the MCI group were *Clostridium_XlVb, Megasphaera, Limosilactobacillus, Lacticaseibacillus, Raoultella*, and *Buttiauxella.* Comparing the AD group to the MCI group, we detected that the relative amounts of *Anaerovorax, Paramuribaculum*, and 2 unidentified genera of the Comamonadaceae and Flavobacteriaceae families increased significantly, whereas *Cetobacterium*, *Raoultella*, and an unidentified genus of the Sutterellaceae family decreased significantly in the AD group.

## DISCUSSION

Differences in the gut microbiome (GM) have been reported between healthy individuals and patients with neurological disorders such as PD and iNPH.^6,7,22^ In AD, growing evidence suggests that GM contributes to disease pathogenesis, with some studies indicating that alterations may already appear in the preclinical stage.^9,23^ Associations between AD and the oral microbiome have also been described.^24^

Our findings show differences in bacterial genera across the CO, MCI, and AD groups. Notably, several alterations appeared at multiple stages of cognitive decline. The most prominent patterns were decreases in *Lacticaseibacillus, Raoultella*, and *Buttiauxella*, already detectable in the MCI group, and increases in Anaerovorax and an unknown genus from the Comamonadaceae family in the AD group. These results suggest a gradual shift in gut microbiome composition along the neurodegenerative continuum. To evaluate their potential as early biomarkers of AD, further research is needed to characterize these relatively unknown genera and clarify their roles within the gut-brain axis.

Alpha diversity, reflecting within-sample bacterial diversity, was assessed using 5 indices: Observed, Chao1, ACE, Shannon, and Inverse Simpson. Observed, ACE, and Chao1 indices showed significant differences between CO and MCI as well as between MCI and AD, but not between CO and AD. In contrast, Shannon and Inverse Simpson indices revealed significant alpha-diversity differences across all three groups.

Beta diversity, reflecting differences in community composition between groups, did not differ significantly between CO and AD or between MCI and AD. No significant difference was observed between CO and MCI either, although the model indicated that arrhythmia and statin use may influence beta-diversity patterns in this comparison.

At the genus level, we identified significant differences between the groups and observed recurring alterations in 5 genera (Fig. 1). *Lacticaseibacillus, Raoultella*, and *Buttiauxella* decreased in the MCI group compared with controls and declined further in the AD group. In contrast, the AD group showed increases in the *Anaerovorax* genus and an unidentified genus from the Comamonadaceae family relative to both CO and MCI. These patterns did not align with findings from a recent study on preclinical AD,^9^ which may reflect differences in diet, lifestyle, or cultural background.

We also observed several genus-level changes that appeared only in specific pairwise comparisons. In the MCI group relative to controls, the genera *Falciporphyromonas, Absicoccus*, and *Ligilactobacillus* increased, whereas *Clostridium_XlVb, Megasphaera*, and *Limosilactobactobacillus* decreased. In the AD group compared with the MCI group *Paramuribaculum* and unidentified genera of Flavobacteriaceae increased, while Cetobacterium and an unidentified genus of the Sutterellaceae family decreased. Independently, the AD group showed a reduction in the *Enterobacter* genus compared with controls.

We also observed inconsistent changes in the genus *Absicoccus*: its abundance increased in the MCI group compared with controls but decreased in the AD group. Although statistically significant, these opposing patterns complicate interpretation. This raises the broader question of whether a genus showing a significant difference between 2 groups (eg, CO vs. AD) should exhibit similar changes in other comparisons. It is also possible that some alterations are reversible, with certain taxa increasing at one stage and declining at another. Given the small sample size, some findings may be spurious. At present, the overall trajectory of microbiome changes across the disease continuum remains unclear, including whether specific alterations occur only at certain stages or lack biological relevance. Further studies are therefore needed.

Among the 5 most significantly altered genera, the most clinically interesting genus was *Lacticaseibacillus*. Taxonomically, it has been an independent genus since 2020, as it was formerly part of the larger genus *Lactobacillus*.^25^ The genus consists of lactic acid-producing species, the most well-known being *L. casei, L. paracasei*, and *L. rhamnosus*. Because our bioinformatics pipeline does not resolve taxa to the species level, the specific species present in our samples remain unknown. Nevertheless, lactic acid-producing bacteria are widely used as probiotics, including in functional food products.^26^

In murine and rodent studies, *L. rhamnosus* has been found to suppress the expression of proinflammatory interleukins and interferons.^27^ *L. rhamnosus GG* was also found to improve cognition and reduce neuroinflammation in mice.^28^ It has additionally been reported to decrease depression in rats by regulating cortisone pathways, increasing neurotransmitters such as serotonin, dopamine, epinephrine, and brain-derived neurotrophic factor, and modulating GM composition.^29^

In a randomized and double-blinded study of MCI patients, *L. rhamnosus GG* strain supplementation did not change microbiome diversity, although it did improve cognitive performance and induced minor changes in the GM composition.^30^ In a double-blinded, placebo-controlled, randomized study, people with MCI received *L. rhamnosus GG-*supplement for 3 months, and their neuropsychological test results improved.^31^ Despite these findings, another human study reported that *L. rhamnosus* was not more effective than a placebo regarding stress, HPA-function, inflammation, or cognitive performance.^32^

In a murine model, *L. casei* improved gut-barrier integrity and reduced colonic and systemic inflammation, along with decreasing oxidative stress in the brain; however, its effects were stronger when combined with a specific dietary-fiber complex rather than used alone.^33^ Another study showed that supplementation with both live and heat-killed *L. paracasei* increased hippocampal serotonin levels and upregulated genes related to neuroplasticity, anti-inflammatory pathways, and antioxidant functions in mice.^34^ In a randomized, double-blind trial in PD patients, a strain of *L. paracasei* alleviated nonmotor symptoms, although it did not alter the gut microbiome composition.^35^

The remaining genera are less well characterized. *Raoultella*, for example, is a relatively unknown histamine-secreting genus composed of low-virulence, biofilm-forming, lipopolysaccharide-containing bacteria.^36^ Although it can cause opportunistic infections in immunocompromised individuals, its overall clinical relevance remains unclear.^37^ Other genera of uncertain significance in our dataset include *Buttiauxella*, *Anaerovorax*, and an unidentified genus within the Comamonadaceae family. A recent meta-analysis reported that these genera were not detected in the microbiomes of PD patients,^38^ raising the possibility that the observed changes may be more specific to AD.

Our independently changing genera *Clostridium_XlVb, Megasphaera, Falciporphyromonas, Ligilactobacillus.* An unidentified genus of the Sutterellaceae family, *Cetobacterium. Paramuribaculum* and *Flavobacterium* are of unknown clinical relevance. The *Enterobacter* genus consists of opportunistic pathogens, some of which exhibit antibiotic resistance.^39^

However, the genus *Limosilactobacillus*, which decreased in the MCI group compared with the CO group, consists of lactic acid bacteria, the most well-known being *L. reuteri*. *L. reuteri* is reported to have protective properties in the gastrointestinal epithelium and has also been associated with immunomodulation.^40,41^

Finding differences between the groups is in line with the literature, which states that gut microbiome-related factors correlate with progressive neurodegeneration. We detected alpha-diversity differences, but no beta diversity. According to a meta-analysis, microbial diversity is less rich in AD patients compared with healthy controls.^23^ However, other preclinical AD studies report that in early AD, diversity may not significantly change.^9^

Our participants reported remarkably similar diets: they consumed comparable amounts of meat, vegetables, dairy, and grain products, and preferred darker rye bread over white bread. Intake of beans, noodles, rice, beer, and alcohol was uniformly low. Given that all participants lived in the same geographical and cultural region in Finland, the lack of dietary differences is unsurprising. The typical Finnish diet is characterized by agricultural products, dairy, and meat,^42^ suggesting that diet, the major potential confounder, was largely eliminated. Previous studies also indicate that adequate nutrition may help mitigate cognitive decline.^43,44^

In our cohort, the ApoE ε4 allele was most common in the AD group, where 78.9% carried at least one ε4 allele. In comparison, 35% of the MCI group and 37.8% of controls were ε4 carriers. ApoE ε4 is a well-established genetic risk factor for sporadic AD, whereas ε2 is considered protective and ε3 is largely neutral.^45^ The higher ε4 frequency in the AD group is therefore consistent with previous literature and supports the validity of our diagnostic grouping.

### Strengths and Limitations

A key strength of this study is the well‑characterized cohort, which included cognitively healthy controls, individuals with MCI, and patients with early‑stage AD, enabling comparisons across different stages of cognitive decline. The groups were similar in age, sex, and dietary habits, reducing major confounders, and the use of detailed food diaries is a notable advantage over many previous studies. To further minimize confounding, individuals with diabetes, known to exhibit gut microbiome alterations, were excluded.^46^

This study also has limitations. Its cross‑sectional design prevents assessment of within‑individual changes over time, and longitudinal studies are needed to confirm the observed patterns. The relatively small sample size, particularly in the MCI and AD groups, limits statistical power and generalizability, underscoring the need for validation in larger cohorts.

Another limitation is the absence of biomarker confirmation for MCI and AD diagnoses. At the time of recruitment (2018 to 2019), fluid biomarkers such as plasma p‑tau217 were not yet available, so classification relied on established clinical and neuropsychological criteria. Although this may introduce some diagnostic heterogeneity, it reflects real‑world clinical practice. Importantly, early‑stage alterations have been detected in this same cohort using independent methods, including eye‑movement analysis and tear‑fluid proteomics,^47–49^ supporting the presence of early disease‑related changes.

Focusing on early‑stage AD and including individuals with MCI allowed us to detect microbiome alterations at the earliest stages of cognitive decline. However, variability in diet, gut health, and sample handling may introduce additional noise, and some genus‑level findings may reflect stochastic variation due to the limited sample size. These results should therefore be interpreted with caution.

### Implications and Future Studies

One of the most consistent findings in this study was the reduced abundance of the *Lacticaseibacillus* genus across multiple group comparisons, suggesting a potential link between lower levels of this genus and cognitive decline. Although these results should be interpreted with caution, they identify *Lacticaseibacillus* as a promising target for further investigation in early-stage AD.

More broadly, our findings support the idea that gut microbiome alterations may emerge early in the disease continuum. Future work should examine whether combinations of microbial taxa, rather than individual genera, could enhance the sensitivity and specificity of microbiome-based biomarkers. Longitudinal studies are particularly important to determine whether these microbial changes precede cognitive decline and could contribute to risk prediction.

The potential clinical utility of microbiome profiling remains an area of interest. If validated in larger cohorts, gut microbiome signatures could complement existing diagnostic tools. Interventional studies, such as dietary modifications or probiotic supplementation, may also help clarify whether modulating the gut microbiome can influence disease-related processes. However, the associations observed here do not imply causality, and the biological and clinical relevance of the identified genera requires further investigation.

## CONCLUSIONS

Our findings indicate that gut microbiome composition is altered in the early stages of cognitive decline, including in individuals with MCI, and that these changes resemble those observed in AD. Several genera, such as *Lacticaseibacillus*, *Raoultella*, and *Buttiauxella*—showed consistent decreases from cognitively healthy individuals to MCI and AD. These results suggest that gut microbiome alterations may emerge early in the disease process and highlight specific genera as potential candidates for biomarker development. However, validation in larger and longitudinal cohorts is needed to determine their reliability and clinical relevance.
